# How A Novel Scientific Concept Was Coined the “Molten Globule State”

**DOI:** 10.3390/biom10020269

**Published:** 2020-02-10

**Authors:** Yutaka Kuroda, Shigeru Endo, Haruki Nakamura

**Affiliations:** 1 Department of Biotechnology and Life Science, Tokyo University of Agriculture and Technology, Tokyo 184-8588, Japan; ykuroda@cc.tuat.ac.jp; 2 School of Science, Kitasato University, Sagamihara, Kanagawa 252-0373, Japan; endo@sci.kitasato-u.ac.jp; 3 Institute for Protein Research, Osaka University, Osaka 565-0871, Japan

**Keywords:** Molten Globule state, Akiyoshi Wada, Oleg B. Ptitsyn

## Abstract

As a tribute to Professor Oleg B. Ptitsyn, we organized an interview with Professor Akiyoshi Wada held in Tokyo in the middle of September 2019. Both Professor A. Wada and the late Professor O. B. Ptitsyn greatly contributed to the field of protein biophysics, and they played leading roles in establishing the concept of the “Molten Globule state” 35–40 years ago. This editorial is intended to recount, as accurately as possible, some episodes during the early days of protein research that led to the discovery of this state, and how this concept was coined the “Molten Globule state” and came to be widely accepted by biophysicists, biochemists, and molecular biologists.

## 1. The Naming and Early Days

Professor A. Wada coined the name “Molten Globule state” during a discussion with Professors O. B. Ptitsyn and C. Crane-Robinson at the International Symposium on Peptides, Polypeptides, and Proteins, held in Galzignano, Padova, Italy, in June 1982. At the time, proteins were considered either folded or unfolded, but several researchers—including the groups of A. Wada and O. B. Ptitsyn—had noticed the presence of a compact, globular, folded state with conserved secondary structures but fluctuating sidechains [1,2]. This state could have been coined as commonly as a “compact denatured state with conserved secondary structure”, but A. Wada, who has a deep appreciation of linguistic and phonemes, proposed the name “Molten Globule state”. Indeed, “melted globule state” would be technically and grammatically perfectly fine, but A. Wada opted for “Molten Globule state”, which did sound better. A. Wada vividly remembers that he asked C. Crane-Robinson whether “Molten Globule state” sounded suitable or not for a native speaker during the Padova meeting.

Apparently, both the A. Wada and O. B. Ptitsyn groups swiftly adopted the term Molten Globule state. The name Molten Globule state first appeared in 1983 in a paper published by M. Ohgushi and A. Wada, describing a compact, partially denatured state of Cytochrome c [3]. It was quickly followed by O. B. Ptitsyn’s 1984 paper, describing the folding of Anhydrase [4]. Afterward, the name, Molten Globule state, was not used in English scientific literature for two years until F. Vonderviszt et al. used it again in 1987, marking the start of its extensive usage [5].

## 2. Why and How Research on A Compact Denatured State with Conserved Secondary Structure Was Initiated

It is sometimes instructive to recall how a scientist, or a group of scientists, are led to initiate a line of research. Let us recall the early motivation for studying a compact denatured state with conserved secondary structures in the laboratory of A. Wada at the University of Tokyo. A. Wada’s study of secondary structures originated at the time when he was a postdoctoral fellow in the laboratory of Paul M. Doty at Harvard University, where he measured the intrinsic viscosity and specific rotation to characterize the helix–coil transition of polyglutamine polypeptides upon pH titration [6].

In retrospect, several aspects of the research on the Molten Globule state appear to be related to the experiments performed in Doty’s laboratory, and this could explain why the Molten Globule state of Cytochrome c was observed despite it appearing only under very un-physiological conditions (an acidic pH below 3 and a high salt concentration over 300 mM). Indeed, pH titration was used for monitoring the pH denaturation of polyglutamine polypeptides, and adding salt was a natural idea for lowering the electrostatic repulsive interactions and trying to stabilize the protein structure.

## 3. Static and Kinetic Molten Globule State 

Records indicate that the name “Molten Globule state” was coined in 1982 during the meeting in Padova, but the phenomenon itself was observed earlier by other groups. Interestingly, the paper published in 1981 by D. A. Dolgikh et al. [1] refers to a paper published in 1976 by K. Kuwajima et al., who discovered the presence of a partially denatured state with conserved secondary structures [2]. To date, Kuwajima’s group was among the first to firmly recognize that a kinetic version of the Molten Globule state appears during the folding of proteins [7]. Indeed, the kinetic version of the Molten Globule state added much to the biological significance of this phenomenon, which would have remained a mere curiosity for protein physicists had it not been observed as an intermediate of protein folding. In the 1990s the Molten Globule state as a folding intermediate became the focus of intensive research, and it is worth noting that this concept appears in today’s biochemistry as well as molecular biology textbooks (e.g., *Molecular Biology of the Cell*, 6th edition, Garland Publishing Inc; Finkelstein and Ptitsyn, *Protein Physics: A Course of Lectures*, 2nd edition, Academic Press, an imprint of Elsevier Science).

## 4. The Molten Globule State and A Vision for Quantitative and Large-Scale Biophysical Measurements

**Figure 1:**
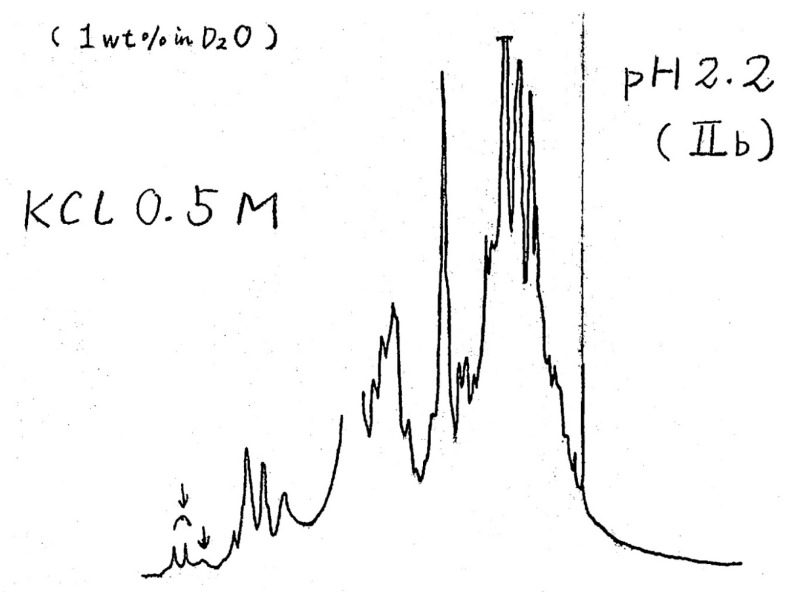
A copy of the original 1D 1H-NMR spectrum of Cytochrome c in the Molten Globule state (initially called state IIb in Wada’s laboratory). The spectrum was measured on December 4th, 1980, with a 360 MHz NMR spectrometer at the Institute for Protein Research, Osaka University. The arrows on the left side show peaks assigned to aromatic protons exhibiting features similar to those in a random coil state (by courtesy of Prof. K. Nagayama).

The research group of A. Wada, which was affiliated to the Department of Physics at the University of Tokyo, was keen to apply modern physical measurements to the analysis of biological systems (for example, Ohgushi and Wada’s paper reports high-resolution NMR spectra of Cytochrome c (Figure 1) as well as quasi-elastic light scattering data, measured by K. Nagayama and K. Soda, respectively). For the study of the Molten Globule state, this meant that the secondary and tertiary structures should be simultaneously monitored with a single sample allowing a quantitative and unambiguous analysis [8]. Practically, a multidimensional measurement system that enabled to measure circular dichroism and fluorescence signals simultaneously on a single sample was used for determining the thermodynamic parameters stabilizing the Molten Globule state of Cytochrome c.

Equipment enabling multi-wavelength measurement is now commonly available, but at the time, they were often home-built. Building such an apparatus certainly necessitates, besides technical expertise, a strong belief, and a vision for the wealth of information that can be deciphered by quantitative and large-scale biophysical measurements. Perhaps it is this vision that led A. Wada to envision the sequencing of the human genome and later initiate structural genomics projects.

## 5. Contribution of Professor Oleg B. Ptitsyn to Protein Biophysics and His Influence in Japan

**Figure 2:**
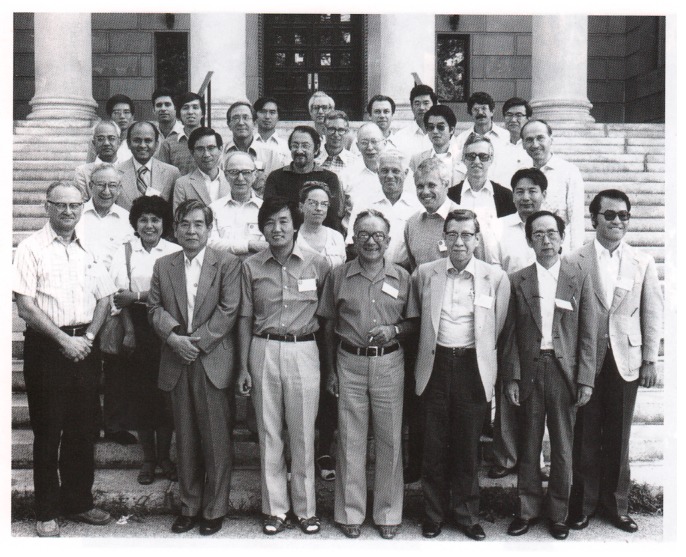
A year before the Padova meeting, O. B. Ptitsyn (first row, fourth from left) and A. Wada (third row, first from left) had met in New York in 1981 during the USA–Japan Seminar on Self-organization Protein Molecules, which was attended by several established Japanese and American protein biophysicists (by courtesy of Prof. A. V. Finkelstein).

O. B. Ptitsyn further developed the concept of the Molten Globule state from a theoretical viewpoint with his colleagues Drs. A. V. Finkelstein and E. Shakhnovich, among many others, and further refined his experimental observation of the Molten Globule states with Dr. V. N. Uversky and many other experimentalists. Professor O. B. Ptitsyn published over 300 research articles, many of which were published in Western journals at a time where communication was cumbersome, and firmly contributed to shaping many aspects of protein biophysics as we know it today. During his endeavor, he met and influenced many Japanese protein scientists on several occasions (Figure 2).

Let us finish this tribute by stating that all appreciated his deep scientific insight, and his kind and warm personality. Here, we reference only publications published during the early days of research on the Molten Globule. We are fully aware that the list of references could be more exhaustive, and we direct interested readers to a recent, more comprehensive review on the Molten Globule concept, which can be found in [9].
